# The yield of chest computed tomography in patients with locally advanced pancreatic cancer

**DOI:** 10.1002/jso.25968

**Published:** 2020-05-06

**Authors:** Mustafa Suker, Bas Groot Koerkamp, Joost J. Nuyttens, Roy S. Dwarkasing, Marjolein Y. V. Homs, Ferry A. L. M. Eskens, Casper H. J. van Eijck

**Affiliations:** ^1^ Department of Surgery Erasmus MC University Medical Center Rotterdam The Netherlands; ^2^ Department of Radiotherapy Erasmus MC University Medical Center Rotterdam The Netherlands; ^3^ Department of Radiology Erasmus MC University Medical Center Rotterdam The Netherlands; ^4^ Department of Medical Oncology Erasmus MC University Medical Center Rotterdam The Netherlands

## Abstract

**Objective:**

To evaluate the incidence of pulmonary metastases on chest computed tomography (CT) in patients with locally advanced pancreatic cancer (LAPC).

**Methods:**

All patients diagnosed with LAPC in a single tertiary center (Erasmus MC) between October 2011 and December 2017 were reviewed. The staging chest CT scan and follow‐up chest CT scans were evaluated. Pulmonary nodules were divided into three categories: apparent benign, too small to characterize, and apparent malignant.

**Results:**

In 124 consecutive patients diagnosed with LAPC, 119 (96%) patients underwent a staging chest CT scan at the initial presentation. In 88 (74%) patients no pulmonary nodules were found; in 16 (13%) patients an apparent benign pulmonary nodule was found, and in 15 (13%) patients a pulmonary nodule too small to characterize was found. Follow‐up chest CT scan(s) were performed in 111 (93%) patients. In one patient with either no pulmonary nodule or an apparent benign pulmonary nodule at initial staging, an apparent malignant pulmonary nodule was found on a follow‐up chest CT scan. However, a biopsy of the nodule was inconclusive. Of 15 patients in whom a pulmonary nodule too small to characterize was found at staging, 12 (80%) patients underwent a follow‐up CT scan; in 4 (33%) of these patients, an apparent malignant pulmonary nodule was found.

**Conclusion:**

In patients with LAPC in whom at diagnosis a chest CT scan revealed either no pulmonary nodules or apparent benign pulmonary nodules, routine follow‐up chest CT scans is not recommended. Patients with pulmonary nodules too small to characterize are at risk to develop apparent malignant pulmonary nodules during follow‐up.

## 1 INTRODUCTION

Projections indicate that pancreatic cancer will be the second leading cause of cancer‐related death by 2030.^1^ At the time of diagnosis, 15% of patients with pancreatic cancer have (borderline) resectable disease (stage I or II), whereas 35% of patients present with locally advanced pancreatic cancer (LAPC; stage III), and 50% of patients initially present with metastatic disease (stage IV).^2^ The definition of LAPC is determined by the extent of tumor contact with the superior mesenteric artery, celiac artery, superior mesenteric vein, and portal vein.^3^ Moreover, imaging should demonstrate no evidence of metastatic disease.

A chest computed tomography (CT) scan is more sensitive and specific in detecting pulmonary metastases than a conventional chest X‐ray.^4^ In patients with pancreatic cancer, the National Comprehensive Center Network (NCCN) guidelines recommend routine chest CT scans.^5^ Chest CT scan in (borderline) resectable pancreatic cancer, nonetheless, was found to be of no influence on survival.^6^, ^7^, ^8^ Chest CT scans frequently reveal subcentimeter pulmonary nodules that are often said to be too small to characterize. They impose a clinical dilemma, as these nodules of uncertain nature induce uncertainty with regard to their nature and as such carry a huge emotional burden to patients. These findings often lead to additional invasive diagnostic tests, which delays the start of treatment and can impose additional risks to the patients. For example, diagnostic transthoracic lung biopsies harbor a considerable risk of pneumothorax or intrathoracic bleeding and frequently are found to be nondiagnostic.^9^

Moreover, the clinical value of a chest CT scan in LAPC could be questioned, because systemic chemotherapy is the first‐line treatment for both LAPC and metastatic disease.^10^ Detection of metastatic disease in LAPC patients is particularly relevant in the era of several locoregional treatments for pancreatic cancer, including radiofrequency ablation, irreversible electroporation, and stereotactic body radiotherapy (SBRT).^11^ While the benefit of these treatments has not been shown definitively, even their strongest proponents agree that they are unlikely to benefit patients with metastatic disease. The aim of this study is to evaluate the yield of routine chest CT scans in patients with LAPC at initial staging and during follow‐up.

## 2 METHODS

We retrospectively reviewed all consecutive patients diagnosed with LAPC between October 2011 to December 2017 seen at Erasmus MC, The Netherlands. The database used for this study was approved by the Institutional Review Board, and informed consent was waived. A diagnostic CT scan of the chest and abdomen was performed at diagnosis and during follow‐up. The CT scan was done on a 128 slice CT scanner with three phases (unenhanced, late arterial [35 seconds], and portal venous [70 seconds]) of the upper abdomen after intravenous injection of contrast medium. In addition, the lower abdomen and chest were scanned in the last phase. The majority of the staging CT scans were performed in our institute; however, some patients already underwent a staging CT scan in the hospital of referral. If the quality of these CT scans was up to the standard and scan were performed less than 4 weeks before therapy, these scans were added in our imaging archive and formally reassessed. Otherwise, the patient underwent a new CT scan in our institute following the guidelines as described above. Diagnosis of LAPC was according to the Dutch guidelines.^12^

All patients with LAPC were offered a treatment consisting of eight cycles of FOLFIRINOX followed by either conventional or stereotactic body radiotherapy when no disease progression was observed on follow‐up scanning. If FOLFIRINOX was not feasible, other chemotherapy regimens or best supportive care were discussed with the patient. Usually, follow‐up CT scans were performed after four and eight cycles of FOLFIRINOX, and 3 months after radiotherapy. In the case of SBRT, an additional CT scan was performed after 6 months. After this, patients underwent CT scans only on the indication. Surgery was offered to patients if induction therapy downstaged the disease to (borderline) resectable pancreatic cancer. The Dutch Pancreatic Cancer Group definitions for resectability were used.^12^

**Figure 1:**
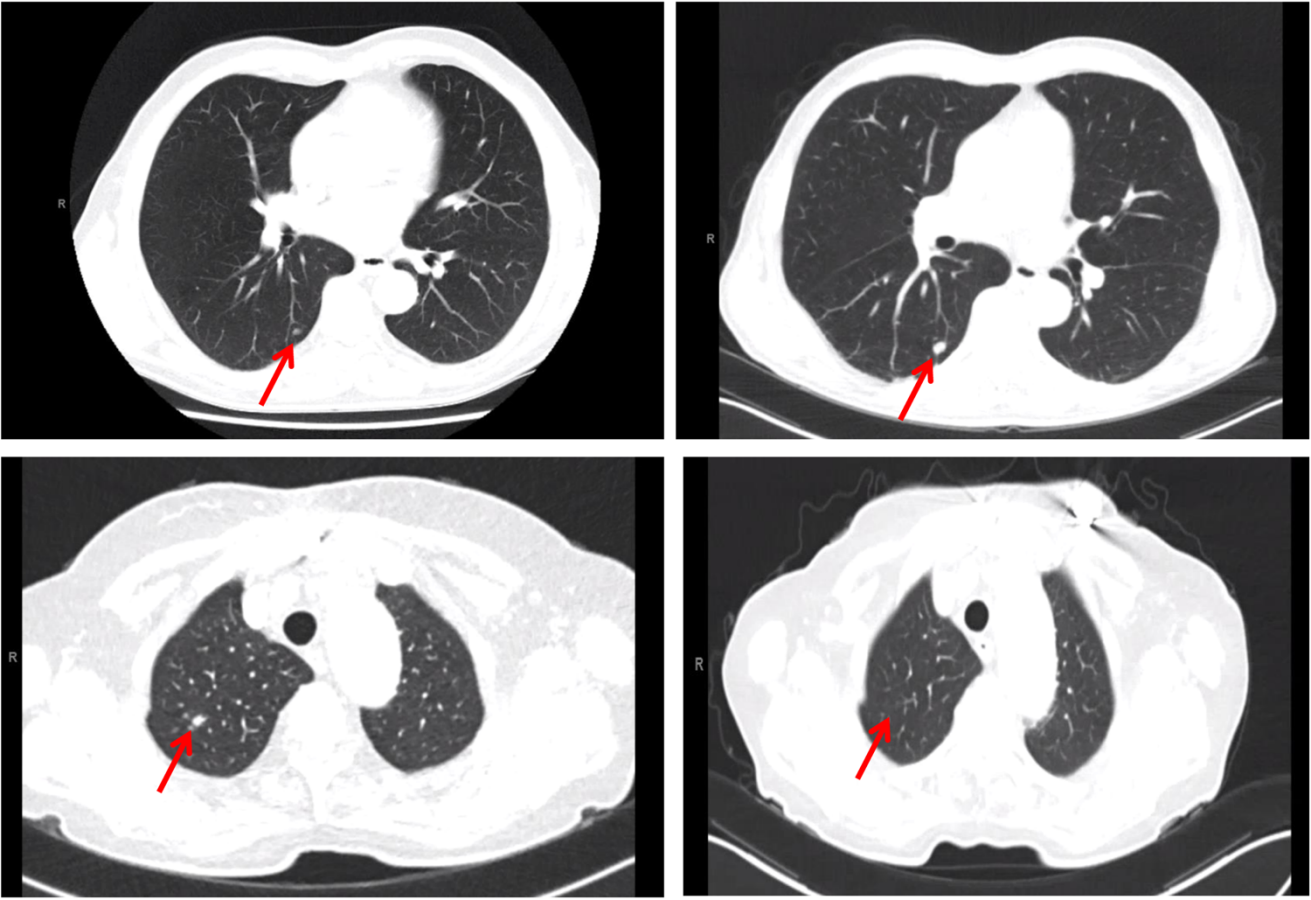
Staging (left) and follow‐up (right) computed tomography scans of patients with nodule too small to characterize (up), and benign nodule (under) [Color figure can be viewed at wileyonlinelibrary.com]

Pulmonary nodules observed during initial and follow‐up CT scans were divided into three categories: apparent benign, too small to characterize, and apparent malignant, whereby an apparent benign nodule was defined as a lesion with homogenous calcification. A nodule was considered too small to characterize was a noncalcified nodule under 1 cm, or pleural effusion (Figure 1).^8^

Comparisons of patient's characteristics between patients without pulmonary nodule or benign nodules versus patients with nodules too small to characterize were analyzed using Fisher exact test for categorical variables, and a nonparametric median test for continuous variables. Overall survival (OS) was calculated from the date of first staging CT scan until the death of any cause. The survival outcome is presented using Kaplan‐Maier and compared logrank in SPSS (version 21). A *P* value less than .05 was considered statistically significant.

## 3 RESULTS

In total 124 consecutive patients diagnosed with LAPC between December 2011 and December 2017 were identified. In 119 (96%) patients (45% male, median age 64 years [interquartile range—IQR, 56‐70]) a staging chest CT scan was available. The World Health Organization's performance score was 0 or 1 in 85 (71%) patients. The tumor was located in the pancreatic head in 73 (61%) of the patients, in the body in 40 (34%) patients, and in 6 (5%) in the tail. LAPC diagnosis was based on arterial contact in 74 (62%) patients, venous contact in 18 (15%) patients, and both venous and arterial contact in 27 (23%) patients. The median baseline serum level of CA19‐9 was 233 [IQR, 61‐974] and of CEA 6.3 [IQR, 3.0‐18.3]. All baseline characteristics are shown in Table 1.

**Table 1:** Baseline characteristics

Baseline characteristics	N = 119 (% or IQR)
Age, median	64 [56‐70]
Sex	
Male	53 (45)
Female	66 (55)
WHO PS	
0‐1	85 (71)
2‐4	34 (29)
Smoking	
Yes	33 (28)
Never	38 (32)
Former	42 (35)
Missing	6 (5)
BMI, median	24 [21‐27]
Tumor origin	
Head	73 (61)
Body	40 (34)
Tail	6 (5)
Maximum tumor size, mm	37 [30‐44]
LAPC based on	
Only arterial	74 (62)
Only venous	18 (15)
Both arterial and venous	27 (23)
Median CA19‐9, µg/L	233 [61‐966]
Median CEA, kU/L	6.3 [3.0‐18.3]

Abbreviations: BMI, body mass index; IQR, interquartile range; LAPC, locally advanced pancreatic cancer; PS, performance status; WHO, World Health Organization.

Best supportive care was initiated in 35 (29%) after the initial diagnosis of LAPC. The reason for initiating the best supportive care was patients' condition in 20 (57%), and patients' requests in 15 (43%) patients. FOLFIRINOX was given as first‐line treatment in 81 (68%) patients, nab‐paclitaxel and gemcitabine in 2 (2%) patients, and gemcitabine alone in 1 (1%) patient. Subsequent radiotherapy was given in 56 (68%) patients after induction chemotherapy. The reason for not receiving radiotherapy after chemotherapy was progression after chemotherapy in 13 (50%) patients, and toxicity in 13 (50%) patients. Conventional radiotherapy was given to 19 (34%) patients, while stereotactic body radiotherapy was given to 37 (66%) patients. Eventually, seven (6%) patients underwent resection.

**Figure 2:**
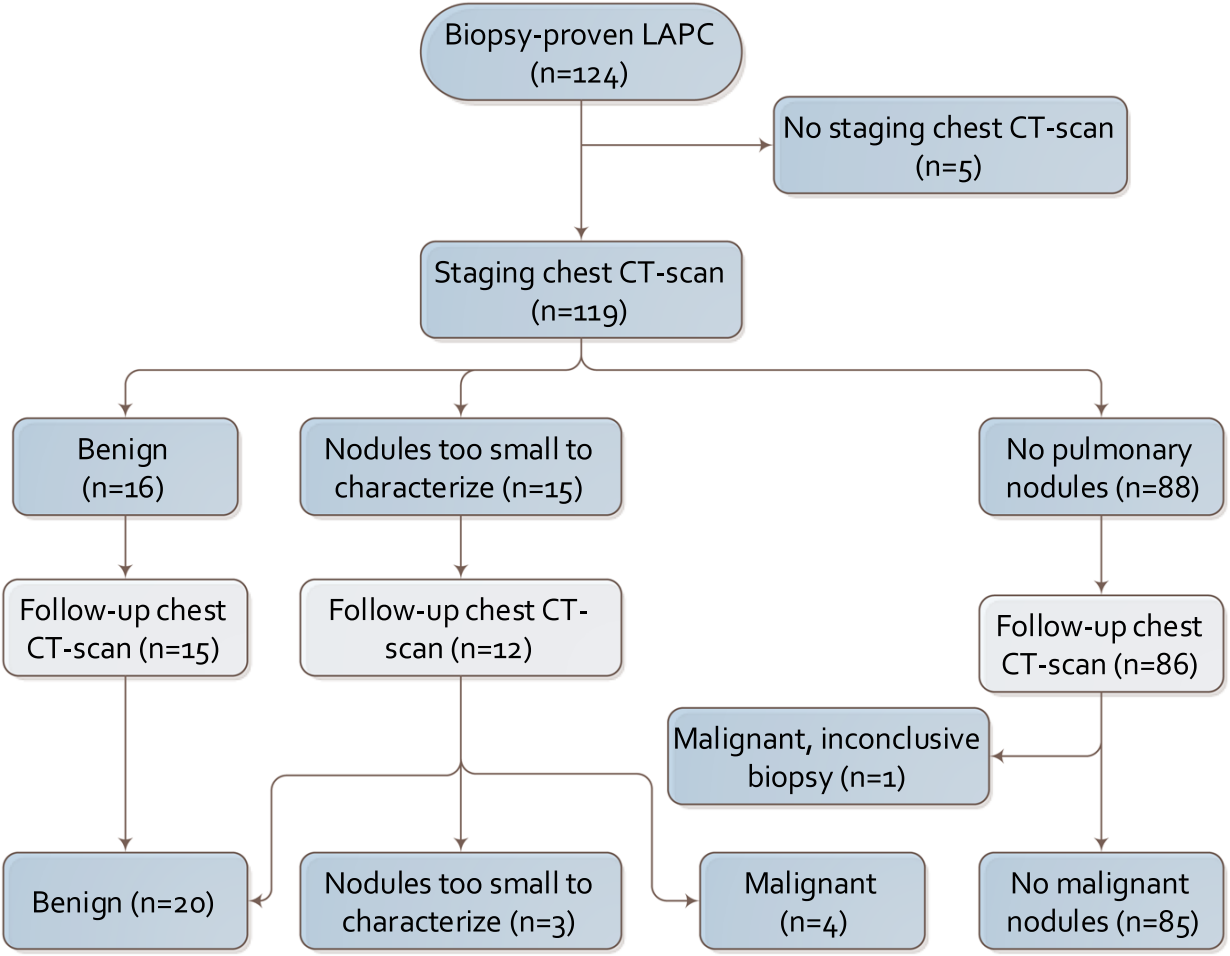
Flowchart of the study population. CT, computed tomography; LAPC, locally advanced pancreatic cancer [Color figure can be viewed at wileyonlinelibrary.com]

In 31 (26%) patients a pulmonary nodule was found on the initial staging CT scan. In 15 (13%) patients the nodules were classified as too small to characterize, whereas in 16 (13%) patients the nodules were classified as apparent benign. The baseline characteristics gender, age, tumor diameter, tumor location, smoking history, and baseline serum CA19‐9 and CEA were not associated with the presence of nodules too small to characterize on staging chest CT scan (Table 2). A follow‐up chest CT scan was performed in 111 (93%) patients (Figure 2), the median time between staging and follow‐up CT scan was 7 months [IQR, 2‐15]. The median number of follow‐up chest CT scans was 2 [IQR, 1‐4]. In one (1%) patient in whom the initial CT scan no pulmonary nodule was seen, malignant appearing pulmonary nodules were seen during follow‐up. The follow‐up chest CT scan was performed for restaging purposes before the start of treatment 1 month after a first chest CT scan. However, a biopsy of one of the nodules was inconclusive. Of the 15 patients in whom the initial CT scan revealed a pulmonary nodule too small to characterize on staging imaging, 12 (80%) patients underwent a follow‐up chest CT scan after a median time of 4 months [IQR, 2‐20]. In four (33%) of these patients, an apparent malignant pulmonary nodule was observed, which coincided in one patient with the development of liver metastasis. Whereas, in five (42%) patients no apparent malignant nodule on follow‐up chest CT scan was found, while three (25%) patients had unchanged nodules. In these patients, no biopsies or resections were performed to obtain a pathological confirmation in any of the radiologically apparent malignant pulmonary nodules. The indication for these follow‐up scans was restaging in nine (75%) patients and deterioration of condition in three (25%) patients. The CT scan of the three patients with deterioration of condition showed local progression in one (33%) patients, liver metastases in one (33%) patients, and liver and peritoneal metastases in one (33%) patient. Clinical characteristics of the patients with nodules too small to characterize on first staging chest CT scan are shown in Table 3.

**Table 2:** Comparing clinical characteristics for patients with and without nodules too small to characterize on staging CT scan

	Patients with nodules too small to characterize (N = 15)	Patients with benign or without pulmonary nodules (N = 104)	*P* value
Age, median [IQR]	68.5 [60.7‐70.1]	63.5 [55.6‐69.8]	.09
Male gender	54%	43%	.58
Smoking (current)	23%	30%	.75
Tumor origin (head)	40%	39%	1.00
Maximum tumor size [IQR], mm	37 [35‐47]	37 [30‐44]	.81
Median CA19‐9 [IQR], µg/L	244 [169‐1392]	231 [56‐966]	.97
Median CEA [IQR], kU/L	5.7 [3.0‐50.5]	6.5 [3.1‐18.0]	.96
Chemotherapy	60%	73%	.36
Radiotherapy	40%	49%	.59
Survival (95% CI), mo	13 (10‐15)	11 (3‐18)	.88

Abbreviations: CI, confidence interval; CT, computed tomography; IQR, interquartile range.

**Table 3:** Clinical characteristic of the patients with nodules too small to characterize on first staging chest CT scan

Patient	Sex	Age, y	Tumor location in pancreas	Baseline CA19‐9	Baseline CEA	First‐line treatment	Radiotherapy	CA19‐9 difference	CEA difference	Pulmonary nodule on follow‐up CT	Progression site	Alive	Survival, mo
1	Female	73	Head	2675	8,0	FOLFIRINOX	SBRT	−2568	−3.29	Unchanged	No	Yes	18
2	Female	74	Head	169	…	BSC	No	…	…	…	…	No	5
3	Male	67	Body	13674	113,0	FOLFIRINOX	No	…	…	Benign	…	No	6
4	Female	64	Head	1392	50,5	BSC	No	…	…	Unchanged	…	No	8
5	Male	68	Tail	11	103,0	FOLFIRINOX	SBRT	+19	−20.3	Malignant	Liver	No	25
											Lung		
6	Female	70	Head	931	4,4	BSC	No	…	…	Benign	…	No	3
7	Male	54	Body	921	5,7	FOLFIRINOX	SBRT	−710	−2.1	Malignant	Lung	Yes	34
8	Male	69	Head	227	…	FOLFIRINOX	No	…	…	Malignant	Lung	No	8
											Local		
9	Male	70	Head	244	1,4	BSC	No	+769	+0.5	Unchanged	Liver	No	6
10	Male	53	Head	…	…	BSC	No	…	…	…	Local	No	13
11	Female	69	Body	…	…	Nab‐paclitaxel + gemcitabine	SBRT	…	…	Malignant	Lung	No	34
12	Female	68	Head	201	2,4	FOLFIRINOX	Conventional	−144	−1.0	Benign	Liver	No	25
											Peritoneum		
13	Female	73	Head	…	…	BSC	No	…	…	…	…	No	11
14	Male	61	Body	150	3,0	FOLFIRINOX	SBRT	+1742	+1.4	Benign	Liver	No	14
											Peritoneum		
15	Male	60	Body	…	…	FOLFIRINOX	SBRT	…	…	Benign	Liver	No	7

Abbreviations: BSC, best supportive care; CT, computed tomography; SBRT, stereotactic body radiotherapy; …, missing data.

**Figure 3:**
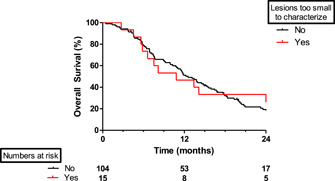
Kaplan‐Meier of patients with and without nodules too small to characterize on first staging chest CT scan (*P* = .88). CT, computed tomography [Color figure can be viewed at wileyonlinelibrary.com]

Median follow‐up time for all 119 patients was 36 months (95% confidence interval [CI], 31‐40), while median OS after first chest CT scan was 12 months (95% CI, 10‐14). There was no difference between patients with benign or without pulmonary nodules vs patients with nodules too small to characterize for receiving chemotherapy (72% vs 60%; *P* = .49) or radiotherapy (49% vs 40%; *P* = .59). The median OS for patients with pulmonary nodule too small to characterize was 11 months (95% CI, 4‐18) vs 13 months (95% CI, 10‐15) in patients without these nodules (*P* = .88) (Figure 3).

## 4 DISCUSSION

Staging and restaging chest CT scans are routinely performed in patients with LAPC. To our knowledge, this is the first study to, retrospectively though, assess and valuate the clinical value of these CT scans, dividing any observed pulmonary nodule into any of three categories: apparent benign, too small to characterize, and apparent malignant pulmonary nodules too small to characterize were seen on first staging chest CT scan in fifteen (13%) patients with LAPC. In this group of patients, follow‐up chest CT scan revealed a subsequent apparent malignant nodule in four patients. Of these four patients, one patient had simultaneous a liver metastasis. Thereby, staging and follow‐up chest CT scan performed in 111 patients gave additional information only in 3 (3%) patients. All the malignant nodules found on follow‐up CT scans were first seen on the staging CT scan as nodules too small to characterize. These findings suggest that follow‐up CT scans are only of clinical value if there is a pulmonary nodule too small to characterize on the first staging CT scan.

In the group with no pulmonary nodules on first staging CT scan, one (1%) patient showed a possible malignant appearing nodule. However, there was radiological uncertainty about this diagnosis. Therefore, the patient underwent a transthoracic biopsy which yielded no confirmation of a malignancy. The patient started with systemic chemotherapy, but stopped after two cycles due to deterioration of condition. No other follow‐up chest CT scan were performed after the restaging CT scan. The patient died eventually 5 months after first chest CT scan, and 2 months after last cycle of FOLFIRINOX. This case gives more insight about the clinical dilemmas of follow‐up chest CT scans in LAPC patients.

The NCCN guidelines advise a staging chest CT scan in all pancreatic cancer patients.^13^ In addition to these guidelines, or maybe to challenge the evidence of them, retrospective observational studies have assessed the added value of chest CT scans in patients with resectable pancreatic cancer.^7^, ^8^, ^14^ Poruk et al^14^ showed that in 183 patients with resectable pancreatic cancer and nodules too small to characterize on the staging CT scan, 16% of the patients subsequently developed apparent malignant pulmonary nodules during routine follow‐up chest CT scans. Nonetheless, there was no difference in median OS between patients with and without these nodules too small to characterize. More recently, Mehtsun et al^7^ showed that in 451 patients with resectable pancreatic cancer with pulmonary nodules too small to characterize, subsequent apparent malignant nodules in was found in only 19 (4%) patients. In this study, there was also no difference in median OS between patients with and without pulmonary nodules too small to characterize. In the LAPC setting, exclusion of metastatic disease is of the essence. Therefore, staging chest CT scan seems reasonable, especially in the era of local therapies emerging as possible new treatment for LAPC.^15^ For treatment monitoring purposes restaging chest CT scans are recommended.^13^ Nonetheless, our study shows that patients without any pulmonary nodule on staging CT scan only one patient developed malignant appearing nodules evidence during follow‐up chest CT scans, without any histopathological proof. Furthermore, only 4% of the patients showed metastatic pulmonary nodules in follow‐up CT scans. These restaging chest scans could be an extra burden for patients, as small nodules could be seen. This could impose additional stress to these patients, as it could also implicate the clinical management. Physicians face the decision to do diagnostics on these nodules or ignore them, keeping in mind that local therapy could be a futile treatment strategy for these patients. In the current study, there was no difference in initial treatment management between patients with and without pulmonary nodules.

The main limitation of our study is its retrospective design, which implicates that patients who were deemed as metastasized pancreatic cancer due to pulmonary metastasis are missed in this study. Moreover, the definitions used for resectability before and after induction therapy are more conservative than used by NCCN guidelines, which could influence the generality of our findings. Furthermore, the data is obtained from only one institute. Nonetheless, our institute is the biggest academic hospital in the Netherlands where most of the patients are referred from nonacademic hospitals. However, more studies are needed to confirm our findings.

In conclusion, follow‐up chest CT scans added information on pulmonary metastasis only in 4% of the patients. However, these nodules were first seen as too small to characterize on staging chest CT scans. The management and survival of patients with nodules too small to characterize on staging CT scan did not significantly differ from patients without these nodules. Routinely follow‐up chest CT should be questioned, unless undefined pulmonary nodules are found on staging chest CT scan.

## SYNOPSIS

Routine follow‐up computed tomography (CT) scans of the chest in locally advanced pancreatic cancer is only of value in patients with nodules on staging chest CT scans.

## Data Availability

Data available on request due to privacy/ethical restrictions.
